# Evaluating the Diagnostic Accuracy of an AI-Driven Platform for Assessing Endodontic Treatment Outcomes Using Panoramic Radiographs: A Preliminary Study

**DOI:** 10.3390/jcm13123401

**Published:** 2024-06-11

**Authors:** Wojciech Kazimierczak, Róża Wajer, Adrian Wajer, Karol Kalka, Natalia Kazimierczak, Zbigniew Serafin

**Affiliations:** 1 Department of Radiology and Diagnostic Imaging, Collegium Medicum, Nicolaus Copernicus University in Torun, Jagiellońska 13-15, 85-067 Bydgoszcz, Poland; 2 Department of Radiology and Diagnostic Imaging, University Hospital No. 1 in Bydgoszcz, Marii Skłodowskiej Curie 9, 85-094 Bydgoszcz, Poland; 3 Kazimierczak Private Medical Practice, Dworcowa 13/u6a, 85-009 Bydgoszcz, Polandnatnowicka@gmail.com (N.K.); 4 Dental Primus, Poznańska 18, 88-100 Inowrocław, Poland

**Keywords:** artificial intelligence (AI), automatic detection, diagnosis, diagnostic test accuracy, orthopantomograms, panoramic radiograph, endodontic treatment

## Abstract

**Background/Objectives:** The purpose of this preliminary study was to evaluate the diagnostic performance of an AI-driven platform, Diagnocat (Diagnocat Ltd., San Francisco, CA, USA), for assessing endodontic treatment outcomes using panoramic radiographs (PANs). **Materials and Methods:** The study included 55 PAN images of 55 patients (15 males and 40 females, aged 12–70) who underwent imaging at a private dental center. All images were acquired using a Hyperion X9 PRO digital cephalometer and were evaluated using Diagnocat, a cloud-based AI platform. The AI system assessed the following endodontic treatment features: filling probability, obturation adequacy, density, overfilling, voids in filling, and short filling. Two human observers independently evaluated the images, and their consensus served as the reference standard. The diagnostic accuracy metrics were calculated. **Results:** The AI system demonstrated high accuracy (90.72%) and a strong F1 score (95.12%) in detecting the probability of endodontic filling. However, the system showed variable performance in other categories, with lower accuracy metrics and unacceptable F1 scores for short filling and voids in filling assessments (8.33% and 14.29%, respectively). The accuracy for detecting adequate obturation and density was 55.81% and 62.79%, respectively. **Conclusions:** The AI-based system showed very high accuracy in identifying endodontically treated teeth but exhibited variable diagnostic accuracy for other qualitative features of endodontic treatment.

## 1. Introduction

Endodontic treatment, commonly known as root canal therapy, is a dental procedure aimed at treating issues within the pulp and root canal system of a tooth. The primary goal of endodontic treatment is to preserve the natural tooth by eliminating infection and preventing future microbial invasion, thereby maintaining the tooth’s functionality and health. Conventional endodontic treatment aims to prevent and eliminate apical periodontitis, which may ultimately lead to tooth loss [1,2]. Moreover, apical periodontitis has the potential to seriously compromise systemic health [3,4].

Successful endodontic treatment relies on thorough disinfection of the root canal system and preventing reinfection through proper instrumentation, irrigation, and obturation [5]. Radiographic evaluation is a critical component of assessing the success of endodontic treatments. The key factors indicating treatment success are the absence of periapical radiolucency and the presence of a well-sealed root canal filling [5]. The success of endodontic treatment is largely influenced by the quality of the root canal filling, with deficiencies in its extent and continuity negatively affecting the treatment outcome [6]. The lack of pathologic radiolucency in follow-up radiographs is a crucial sign of successful treatment [7].

Due to their high diagnostic accuracy, low cost, and accessibility, periapical radiographs (PRs) are the preferred treatment modality for endodontic assessment [5]. However, due to their limited field of view (FOV), PRs are insufficient for comprehensive evaluation of dental status. Panoramic X-rays (PANs) overcome this limitation by providing a broad view of the orofacial region, including all teeth, the maxilla, the mandible, and surrounding structures such as the temporomandibular joints. Therefore, PAN is the most frequently prescribed radiographic examination in dentistry [8]. PAN is a widely used imaging technique in dental practice due to its ability to provide a comprehensive view of the maxillofacial region. However, in endodontic treatment, PRs are preferred due to their higher resolution and accuracy in detecting fine details such as minor canal overfillings. PRs are often taken immediately after PAN to complement the initial assessment. PAN is utilized in numerous practices and has been shown to be useful in different applications, including orthodontics, surgery, and periodontology [9]. However, its role in endodontic assessment, particularly in diagnosing periapical lesions and other endodontic conditions, remains controversial due to its limited diagnostic accuracy [10,11]. Therefore, PAN has been used mainly in large epidemiological surveys assessing large cohorts of patients and as a basic screening test [12].

The recent surge in artificial intelligence (AI) applications in medicine has extended to dentistry, particularly in dentomaxillofacial radiology [13,14]. AI technologies, including machine learning (ML) and deep-learning DL, show promise in enhancing diagnostic accuracy and reducing diagnosis time. AI has already been tested for various applications in dentomaxillofacial radiology, including caries detection, periodontal and orthodontic evaluation, cysts and tumors detection, among others [15]. The increased radiological exam volumes and practitioner workloads have led to the development of commercially available AI tools to aid diagnostics. Diagnocat Ltd. (San Francisco, CA, USA) has created a system using convolutional neural networks (CNNs) trained on over 35,000 dental radiographs for accurate dental diagnostics. The system is capable of making detailed diagnoses, including the periodontological, orthodontic, and endodontic status of the patient. Theoretically, it could aid in a prompt and accurate diagnosis. However, the current body of literature still lacks studies assessing the diagnostic accuracy of various modules of the program.

The purpose of this preliminary study was to evaluate the diagnostic performance of an AI-driven platform, Diagnocat, in assessing radiographic endodontic treatment outcomes using PAN images. This evaluation aimed to determine the accuracy and reliability of the AI system compared to the consensus of two observers.

## 2. Materials and Methods

The study was conducted in accordance with the Declaration of Helsinki and approved by the Ethics Committee of Collegium Medicum, Nicolaus Copernicus University in Torun, Poland (protocol No. KB 227/2023, 10 April 20223), for studies involving humans.

### 2.1. Patients

The study involved 55 patients (15 males and 40 females, aged 12–70) who were referred for PAN imaging at a private dental center. Referrals were made by orthodontists and dental surgeons between January and September 2023. PAN images were prescribed as the initial imaging modality. The primary exclusion criteria were poor overall image quality and motion artifacts. 

### 2.2. Image Acquisition and Postprocessing

All PAN images were captured using a Hyperion X9 PRO digital cephalometer (Myray, Imola, Italy). To ensure patient anonymity, identifiers were removed, and images were coded for blinded analysis. After image acquisition, no manipulations (filters, cropping) were performed to avoid impacting the results of the assessments.

### 2.3. AI Evaluation

PAN images were manually uploaded to Diagnocat, a cloud-based AI platform (Diagnocat Ltd., San Francisco, CA, USA). The AI software automatically generated reports estimating the probabilities (0–100%) of various conditions. A probability of 50% was considered a positive diagnosis.

For endodontic treatment assessments, the AI evaluated the following:

1. Probability of filling;
2. Adequate obturation;
3. Adequate density;
4. Overfilling;
5. Voids in filling;
6. Short filling;
7. Root canal number.

### 2.4. Evaluation of Human Readers

The images were independently evaluated by two observers, one general dentist and one radiologist, with 5 and 8 years of experience, respectively. The reading sessions were conducted on a specialized console using iRYS Viewer software version 6.2 from MyRay, Italy. All images were reviewed on a medically certified monitor RadiForce MX243W (Eizo, Hakusan, Japan).

The presence of root canal fillings and the features listed in the AI reports were recorded for each tooth. The following criteria were applied during the assessment:

- Adequate filling is defined as the root canal filling extending to 0–2 mm from the radiographic apex without voids; consistency and density of fillings were evaluated;
- Adequate density is characterized by homogenous radiopacity along the length of the root canal filling, indicating complete obturation;
- Overfilling is the presence of endodontic material beyond the tooth’s apex;
- Voids in filling are the radiolucent areas within the filling;
- Short filling is defined as the filling extending to less than 2 mm from the radiographic apex.

Each observer assessed each radiograph separately and independently (without knowledge of the AI results or the other reader’s evaluation). After the evaluation, the observers discussed the results, jointly evaluated the images, and reached a consensus, which was considered the reference standard. To assess the reliability of the AI diagnostic reports, they were compared to the consensus reached by the observers.

### 2.5. Statistical Evaluation

To evaluate the accuracy of the AI results, sensitivity, specificity, accuracy, positive predictive value (PPV), negative predictive value (NPV), and F1 score were calculated. Qualitative variables were reported as absolute and relative frequencies (N and %). The diagnostic accuracy calculations were calculated according to the method presented in the paper by Hicks et al. [16]. Analyses were conducted using R software, version 4.4.0.

## 3. Results

### 3.1. Patients

The study included 55 PAN images from 55 patients, with a mean age of 44.53 years (range 12–70 years). Among them, 40 females (mean age 42.83 years, SD 13.42) and 15 males (mean age 50.73 years) were studied. A total of 1330 teeth were analyzed.

### 3.2. Diagnostic Accuracy Parameters

**Figure 1:**
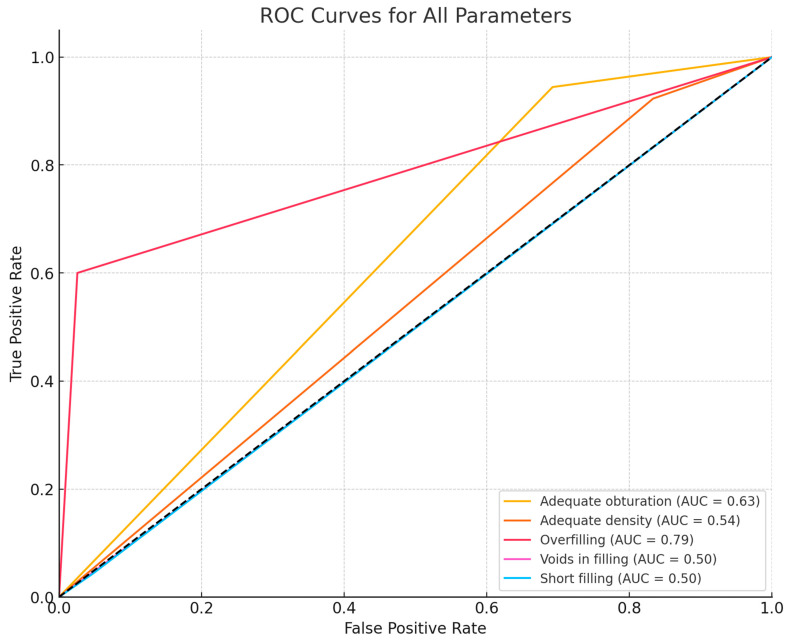
ROC curves for all the assessed endodontic treatment parameters.

The study showed mixed results regarding the diagnostic accuracy of various root canal treatment features assessed by the AI program. Table 1 provides detailed results of the diagnostic accuracy for various endodontic filling features. Figure 1 presents ROC (receiver operating characteristics) curves for all the assessed parameters.

**Figure 2:**
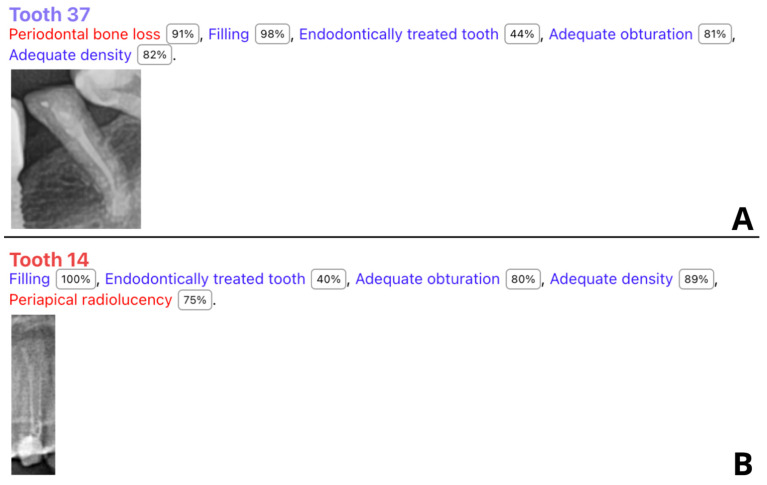
Two (out of ten) sample teeth diagnosed with probability of endodontic treatment below 50% (**A**,**B**).

**Figure 3:**
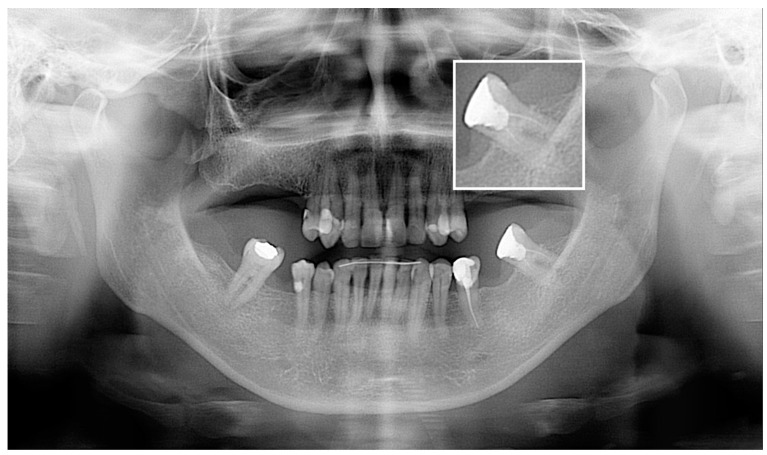
Case of tooth enumerated by Diagnocat (Diagnocat Ltd., San Francisco, CA, USA) as 37 and by the observers as 38.

The AI system demonstrated high accuracy (90.72%) in detecting the probability of filling. The F1 score was 95.12%, indicating a strong balance between sensitivity and specificity. A total of 10 of the 11 false negative cases were correctly identified by the tested software; however, the program indicated a probability of filling below 50%, which was regarded as a negative diagnosis. Figure 2 shows two samples of correctly identified cases of endodontic treatment with assessed endodontic features but with a probability of filling less than 50%. One tooth was misinterpreted by the AI as 37, whereas the observers labeled it 38 (Figure 3). However, the remaining results showed lower accuracy metrics with an unacceptable F1 score for short filling and voids in filling assessments. 

## 4. Discussion

The findings of this study highlight the potential and limitations of an AI-based system for evaluating root canal treatments via PAN images. Our results showed that the AI system demonstrated high diagnostic accuracy for certain features, such as the probability of filling, but had variable performance across other categories.

Previous studies have demonstrated the efficacy of AI in dental radiology, particularly in identifying periapical lesions and other endodontic conditions. For instance, Issa et al. [17] reported the high diagnostic accuracy of AI in detecting periapical lesions (PLs) on PRs, with an emphasis on the ability of AI to enhance diagnostic workflows by reducing human error. Similarly, Orhan et al. (2020) reported that AI systems could effectively identify PLs in cone-beam computed tomography (CBCT) scans, highlighting the potential for AI to support clinical decision-making in endodontics [18]. Similar results were shown in our 2024 study on the diagnostic accuracy of Diagnocat for PL detection in the PAN and CBCT images of one patient cohort [19]. The sensitivity of the AI for PL detection via PAN was 33.33%, with significantly higher metrics for CBCT (77.78%). Another study using a U-Net architecture for CBCT images achieved an even greater lesion detection accuracy of 93%, highlighting the potential of AI in more complex imaging modalities [20]. AI models have demonstrated high sensitivity and precision in various applications, including teeth detection and segmentation, with sensitivity and precision values exceeding 95% [21,22,23]. AI systems exhibited similarly high accuracy in identifying crowns, implants, and impacted teeth, with sensitivity and precision values often above 90% [24,25]. However, AI’s diagnostic accuracy in specific tasks, such as caries or calculus detection, was unsatisfactory, with values below 50% [24,26,27]. These studies show areas for further improvement, and based on current interest and spending related to AI development, these results will soon significantly improve. It should also be noted that despite some inaccuracies, these systems are highly time-effective, offering substantially shorter analysis times and reducing the workload of practitioners [21,23].

Our study’s findings align with these results to some extent, particularly in terms of the AI’s ability to accurately assess the probability of filling with high accuracy. The AI system achieved an accuracy of 90.70%, a specificity of 100%, and a sensitivity of 90.70% for this category, resulting in a strong F1 score of 95.12%. This suggests that the AI system is reliable for identifying cases of endodontic treatment. However, we believe that we have identified some problems with the AI system. Our findings show that the AI program indicated that the probability of filling in 10 patients was less than 50%; however, the AI program also assessed endodontic filling features (Figure 2). Moreover, the program did not exhibit any false positive diagnoses, indicating its high conservativeness in filling probability assessments. Some minor adjustments to the program’s settings might resolve this issue and result in even better diagnostic accuracy metrics. This issue also influenced other parameters, mainly “short filling” assessments. ROC curves showed that the area under the curve (AUC) for overfilling assessment is high, suggesting the model has good discriminative power for this parameter. However, the assessment of voids in filling and short filling features showed low AUC values, indicating poor AI program performance.

**Figure 4:**
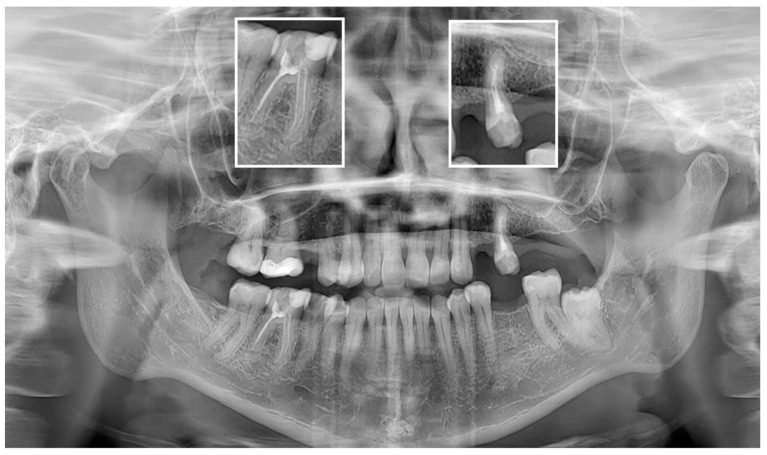
Typical case of false negative short filling diagnosis of teeth 25 and 46.

**Figure 5:**
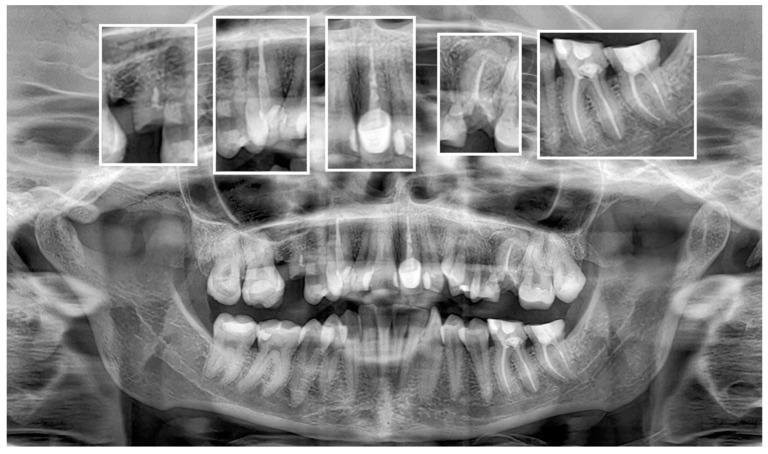
Sample misdiagnoses made by AI program compared to observers’ consensus. Tooth 15—false positive adequate obturation and density; false negative—short filling. Tooth 13—false positive—adequate density. Tooth 21—false positive adequate obturation; false negative—voids in filling, short filling. Tooth 26—false positive adequate obturation; false negative—short filling. Tooth 36—no misdiagnoses. Tooth 37—false positive adequate obturation and density; false negative—voids in filling, short filling.

However, the AI system’s performance was less impressive in other categories. For example, the accuracies for detecting adequate obturation and adequate density were 55.81% and 62.79%, respectively. These results are comparable to those reported by Vujanovic and Jagtap [28], who noted that while AI could detect a high proportion of dental anomalies, its performance varied significantly across different conditions. Similar results were shown by Zadrożny et al. [27], who reported unsatisfactory diagnostic accuracy in endodontic treatment assessment on PAN images. Although the sensitivity for endodontic treatment detection was relatively high (87.2%), the system showed lower sensitivity for over- and underfilled canals, and the homogeneity of canal filling assessments ranged between 45.5% and 60.9%. It should be noted, however, that the study evaluated only 30 PAN images, and as for the date of preparation of the manuscript (May 2024), it allows for a more detailed evaluation with a greater number of features assessed. To the best of our knowledge, no other studies to date have assessed the diagnostic accuracy of Diagnocat for endodontic treatment evaluation. Our study showed results similar to Zadrożny’s, specifically the very low F1 values for the extent of endodontic filling. The short filling features were not correctly identified by AI in most cases. This indicates a lack of proper recognition of tooth apex positions by the AI program. Figure 4 shows typical cases of short filling misdiagnosis. The diagnostic accuracy of the tested AI program exhibited moderate results for the rest of the assessed parameters. Figure 5 presents a sample case with multiple endodontically treated teeth with some misdiagnosed features. 

Although PAN is a widely used imaging technique in dental practice due to its ability to provide a comprehensive view of the maxillofacial region, its diagnostic accuracy, especially in endodontic evaluations, has been a subject of debate. It should be noted that PAN is not the preferred modality in endodontic treatment assessment, with more suitable modalities, such as PR and CBCT [5,29]. PR remains the first-choice modality endodontic treatment assessment due to its superior resolution, accessibility, and low radiation dose [30]. PAN imaging suffers from inherent limitations such as lower resolution, image distortion, uneven magnification, structures’ superimposition, and limited specificity [31]. PAN radiographs have a lower resolution compared with intraoral periapical radiographs [32]. This can result in less detailed images, making it challenging to detect fine details such as minor canal overfillings, which are crucial in endodontic assessments. Furthermore, image distortion and magnification can affect the accuracy of measurements and the spatial relationship of structures, potentially leading to misdiagnosis. The superimposition of anatomical structures, such as root canal fillings, may ultimately prevent correct diagnosis or complicate the interpretation of the radiograph, especially for inexperienced clinicians. Several studies have evaluated the diagnostic accuracy of PAN in detecting various dental conditions, including endodontic lesions and PLs. For instance, one study found that PAN had good diagnostic accuracy (71.3%) and high specificity (93.8%) but low sensitivity (48.8%) for detecting endodontically treated AP when compared with CBCT, which served as the reference standard [11]. All these limitations finally affect the interobserver variability in the interpretation of radiographs, which may impact the consistency of diagnostic accuracy. Therefore, our results should be considered with caution, and in an ideal scenario, all the results should be compared with the results of the reference method evaluation—CBCT. A comparison of the analysis results of PAN images by human observers and AI with the CBCT evaluation would, in fact, allow for an objective assessment of Diagnocat’s diagnostic accuracy of endodontic assessment.

A recent systematic review on the diagnostic and prognostic accuracy of AI in endodontic treatment by Korabari et al. [33] showed that the use of AI could enhance treatment plans and possibly lead to higher success rates in endodontic treatment outcomes. The authors also demonstrated that AI could assist in numerous clinical applications, such as detecting root fractures and PL, determining working length, tracing the apical foramen, understanding root morphology, and predicting diseases. Unfortunately, the authors did not evaluate the efficacy of AI in the radiographic assessment of endodontic treatment results. However, despite these positive results, the authors concluded that meticulous research is still necessary before seamlessly integrating AI systems into everyday practice. Similar results were shown in a few other reviews evaluating AI applications for endodontic treatment [34,35]. These findings fit into the broader context of AI applications in radiology and medicine, where caution is recommended against the uncritical and inadequately researched application of AI systems [36].

This study has several limitations that should be considered. The sample size, while sufficient for preliminary analysis, may not capture the full variability in clinical practice. The sample size was not calculated a priori; instead, it was based on the number of available cases meeting the inclusion criteria within the study timeframe. Additionally, the performance of the AI system may be influenced by the quality and resolution of the panoramic radiographs, which can vary between different imaging systems and settings. Another limitation is the possible impact of human error and the subjectivity of the qualitative assessments on the results of the study. However, to reduce its impact, the two-observers consensus was regarded as the reference standard. There is also a geographical limitation, as all the images were acquired in one dental center with a homogenous population.

Future research should focus on expanding the sample size and including a more diverse patient population to validate the findings. Moreover, integrating AI assessments with other diagnostic tools, such as CBCT, may enhance the overall accuracy and reliability of endodontic evaluations.

## 5. Conclusions

Overall, the AI-based system showed varying levels of performance across different categories. It performed exceptionally well in identifying the probability of filling, with high accuracy, sensitivity, specificity, and F1 score. However, its performance in other categories such as adequate obturation, adequate density, and overfilling varied, with lower accuracy. Taking into account that PAN images are not the preferred imaging modality in endodontic assessments, we consider Diagnocat a very valuable tool in post-endodontic treatment assessments, facilitating prompt and accurate diagnoses. However, our findings highlight the need for further improvement and validation of the AI system to ensure reliable and accurate assessments in clinical practice.

## Figures and Tables

**Table 1:** Detailed diagnostic accuracy metrics.

Parameter	Sensitivity	Specificity	Accuracy	PPV	NPV	F1
Filling probability	90.70%	100.00%	90.70%	100.00%	0.00%	95.12%
Obturation adequacy	94.12%	30.77%	55.81%	47.06%	88.89%	62.75%
Density adequacy	96.00%	16.67%	62.79%	61.54%	75.00%	75.00%
Overfilling	60.00%	97.37%	93.02%	75.00%	94.87%	66.67%
Voids in filling	11.11%	88.24%	72.09%	20.00%	78.95%	14.29%
Short filling	4.35%	100.00%	48.84%	100.00%	47.62%	8.33%

PPV—positive predictive value; NPV—negative predictive value.

## Data Availability

Data are available on request.
